# Radiographic Posterior Tibial Slope Measurement in Medial Unicompartmental Knee Arthroplasty: A Retrospective Validation Study Using Digital Volume Tomography of Tibial Resectates

**DOI:** 10.3390/jcm15062095

**Published:** 2026-03-10

**Authors:** Dimitrios Tsimopoulos, Patrick Ostheim, Moritz Kaiser

**Affiliations:** 1 Department of Radiology, University Hospital Regensburg, 93053 Regensburg, Germany; patrick.ostheim@klinik.uni-regensburg.de; 2 Department of Trauma Surgery, Regensburg University Medical Center, 93053 Regensburg, Germany; moritz.kaiser@mvzkaiser.de

**Keywords:** posterior tibial slope, unicompartmental knee arthroplasty, radiographic measurement, digital volume tomography, tibial alignment

## Abstract

**Background/Objective:** Accurate assessment of the posterior tibial slope (PTS) is essential for optimal alignment and kinematic restoration in unicompartmental knee arthroplasty (UKA). This study aimed to evaluate the accuracy of three commonly used radiographic PTS measurement techniques—the anterior tibial cortex (ATC), tibial proximal anatomical axis (TPAA), and posterior tibial cortex (PTC)—by comparing them with the intraoperatively achieved tibial resection slope, using digital volume tomography (DVT) of intraoperative tibial resectates as an executed resection reference. **Methods:** In this retrospective study, 39 patients undergoing medial UKA were analyzed. Standardized lateral knee radiographs were used to measure the complement angle β using ATC, TPAA, and PTC reference axes. Intraoperatively obtained tibial resectates were scanned using DVT to provide a high-resolution three-dimensional reference. The conventional posterior tibial slope was defined as α (PTS) = 90° − β (measured angle). Agreement and systematic bias between radiographic and DVT measurements were assessed using Wilcoxon signed-rank tests and Bland–Altman analyses. **Results:** The mean DVT-derived β was 86.48° ± 1.62° (α 3.52°). ATC 79.69° ± 3.14° (α 10.31°) and TPAA 82.50° ± 2.95° (α 7.50°) yielded significantly lower β values than DVT (both *p* < 0.0001), whereas PTC (86.24° ± 2.51°; α 3.76°) showed no significant difference (*p* = 0.419). Bland–Altman analyses demonstrated minimal bias for PTC (−0.25°) compared with larger negative biases for ATC (−6.79°) and TPAA (−3.99°) (negative bias indicates lower β and therefore higher conventional posterior tibial slope α). **Conclusions:** Among the evaluated methods, the PTC technique most accurately reflects the intraoperatively achieved tibial resection slope when benchmarked against DVT measurements. Incorporating the PTC method into preoperative planning may improve the radiographic estimation and standardization of the achieved tibial cut in UKA. Further studies should assess its impact on clinical outcomes and explore integration into automated measurement workflows.

## 1. Introduction

Unicompartmental knee arthroplasty (UKA) is an established treatment option for isolated compartmental knee osteoarthritis and may offer advantages over total knee arthroplasty, including less invasive surgery, preservation of bone stock and ligaments, faster recovery, and good functional outcomes in appropriately selected patients [1,2,3]. However, UKA remains technically demanding, and accurate component positioning is essential for implant longevity and optimal clinical results [4,5,6].

Among alignment parameters, the sagittal orientation of the tibial component—commonly expressed as the posterior tibial slope (PTS)—plays a major role in knee biomechanics by influencing tibiofemoral kinematics, cruciate ligament loading, tibial shear forces, and contact pressures [7,8,9]. Small slope deviations can meaningfully alter anterior tibial translation and knee stability, which is particularly relevant in UKA where cruciate ligaments are preserved and restoration of near-physiological motion is a key goal [8,10,11]. Experimental and clinical studies suggest that slope modification affects kinematics and loading patterns, and postoperative slopes above approximately 7° have been associated with inferior pain outcomes, while slopes within a physiological range (often ~4–7°) may support better flexion after medial UKA [4,12,13,14,15,16,17].

Preoperative evaluation of the PTS is most commonly performed using standard lateral knee radiographs, owing to their accessibility, low cost, and suitability for serial comparison [18,19]. Several methods for defining the reference axis on X-rays have been proposed, including the proximal tibial anatomical axis, the tibial shaft line, and the posterior tibial cortical line [20,21]. These methods differ in reliability depending on image quality, tibial length included, and patient positioning [22]. Studies comparing lateral radiographs with CT or long-leg imaging have shown good agreement when radiographic acquisition is standardized, and the anatomic axis is defined from at least 100 mm of the tibial shaft [23,24,25]. In this study, we compared three commonly used radiographic reference methods, the anterior tibial cortex (ATC), the tibial proximal anatomical axis (TPAA) and the posterior tibial cortex (PTC) with the executed tibial resection slope measured on intraoperatively obtained tibial resectates using digital volume tomography (DVT). DVT-based measurements offer high spatial resolution and three-dimensional assessment compared with conventional radiographs. Because the resected tibial segment can be scanned without soft-tissue interference and with a clearly defined cut surface, DVT enables a precise quantification of the intraoperatively achieved tibial resection slope and can serve as a specimen-based executed resection reference for validating radiograph-derived measurement techniques against the executed tibial cut. The aim of this study was to identify the radiographic technique (ATC, TPAA, or PTC) that most accurately reflects the executed tibial resection slope achieved during medial UKA, using ex vivo DVT of the tibial resectate as the specimen-based reference standard.

## 2. Materials and Methods

### 2.1. Study Design and Patient Selection

This retrospective, single-center observational study investigated the preoperative assessment of the tibial sagittal slope in patients undergoing unicompartmental knee arthroplasty (UKA). All consecutive patients who underwent primary UKA for isolated medial compartment osteoarthritis at an institution, that is affiliated with the Regensburg University Medical Center, Germany between October 2019 and March 2020, were screened for inclusion.

A total of 39 patients met the inclusion criteria and were included in the final analysis. Inclusion criteria comprised:

- Primary medial UKA performed for symptomatic isolated medial compartment osteoarthritis after failure of non-operative management with preserved lateral and patellofemoral compartments.
- Availability of a preoperative standardized weight-bearing lateral knee radiograph acquired as part of routine care.
- Radiographs meeting predefined quality criteria: true lateral projection with complete overlap of the posterior femoral condyles (minimizing rotational error) and inclusion of ≥15 cm tibial shaft distal to the joint line (stable definition of tibial axis).
- Availability of the intraoperatively resected tibial bone block (resectate) with intact cut surface and sufficient residual native plateau to allow tangent definition in multiplanar reformats.
- DVT dataset of the specimen with sufficient image quality for multiplanar reformatting and measurement of the resection plane.

Exclusion criteria included any history of tibial fracture, previous high tibial osteotomy, ligament reconstruction, inflammatory joint disease, or inadequate imaging quality.

Demographic data including age, sex, affected side, and diagnosis were collected from the institutional surgical database. All patient data were pseudonymized prior to analysis to maintain confidentiality.

The study was conducted in accordance with the Declaration of Helsinki and approved by the institutional ethics committee (approval number: 25-4140-104).

### 2.2. Imaging and Specimen Preparation

Preoperative imaging consisted of standardized weight-bearing lateral knee radiographs acquired at approximately 30° of knee flexion. Radiographs were accepted for analysis only if they met predefined quality criteria: complete overlap of the posterior femoral condyles (to minimize rotational error) and inclusion of at least 15 cm of the tibial shaft distal to the joint line (to allow a stable definition of the tibial axis) [26]. Images were acquired using a calibrated digital radiography system and exported in DICOM format for analysis.

During surgery, the medial tibial bone resection specimen (resectate) was collected immediately after the tibial cut, labeled, and stored in 10% neutral buffered formalin until imaging. Formalin fixation has been reported to cause only minor dimensional changes in bone specimens compared with other fixation methods, supporting its use for ex vivo geometric assessment [27]. As bone geometry is comparatively stable, we expected negligible impact on angular measurements. Specimens were subsequently scanned using DVT (cone-beam CT), providing a high-resolution three-dimensional dataset suitable for multiplanar reformatting and angular measurements [28,29]. This enabled a direct comparison between the preoperative radiographic slope and the actually executed tibial resection slope derived from the specimen-based DVT dataset.

### 2.3. Preoperative Measurement of the PTS on Lateral Knee Radiographs

There have been, to date, several methods for measuring the PTS on lateral knee radiographs. Various reference-axis–based measurement techniques have been proposed, each producing slightly different slope values depending on the chosen tibial reference line. Dejour and Bonnin define the proximal tibial anatomical axis (TPAA) by connecting the midpoints of the anterior and posterior tibial cortices at 5 cm and 15 cm below the joint line; the slope is then measured as the angle between the tibial plateau tangent and a line perpendicular to this axis [30]. Subsequent approaches have employed alternative reference axes, including the anterior tibial cortex (ATC), posterior tibial cortex (PTC), and long diaphyseal (anatomical) axis, to improve reproducibility [31,32]. To further standardize the measurement and enhance correlation with computed tomography (CT), Utzschneider et al. introduced the mean proximal anatomical axis (MPA) method, which averages the ATC and TPAA axes and demonstrated superior consistency across observers [33].

**Figure 1:**
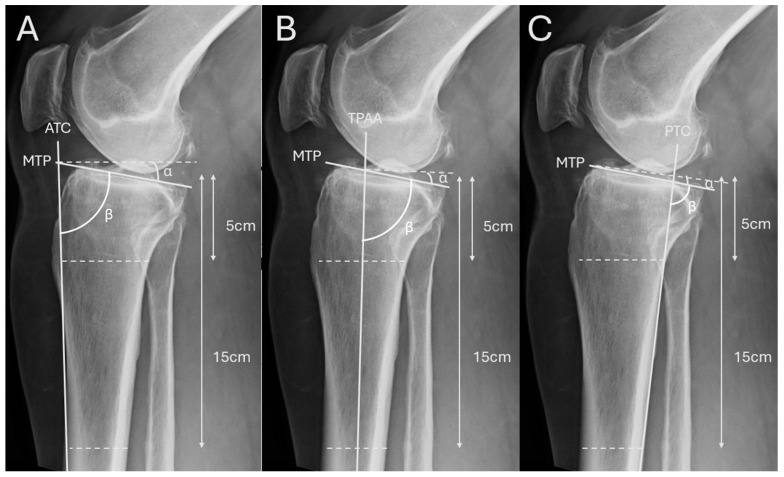
Radiograph-based measurement methods of the PTS. (**A**) Anterior Tibial Cortex (ATC) method: The anterior tibial cortex serves as the reference axis. A line is drawn along the anterior cortical border of the tibia, and the medial tibial plateau (MTP) line is placed tangentially to the joint surface. The PTS is defined by the angle (α) between the MTP and a line perpendicular to the ATC. Angle β is measured between MTP and the reference axis ATC. Reference points are located 5 cm and 15 cm distal to the tibial plateau. (**B**) Tibial Proximal Anatomical Axis (TPAA) method: The proximal tibial anatomical axis (TPAA) represents the anatomical shaft axis of the proximal tibia. To define it, transverse lines are drawn between the anterior and posterior cortices at two levels (5 cm and 15 cm distal to the plateau), and their midpoints are connected to form the TPAA. The angle (α) between the MTP and a line perpendicular to the TPAA corresponds to the PTS. Angle β is measured between MTP and the reference axis TPAA. (**C**) Posterior Tibial Cortex (PTC) method: The posterior tibial cortex is used as the reference axis. A line is drawn along the posterior tibial border, and the PTS is measured as the angle (α) between the MTP and a line perpendicular to the PTC. Angle β is measured between MTP and the reference axis PTC. As in the previous methods, reference distances of 5 cm and 15 cm distal to the tibial plateau are used for axis definition.

Adjacent to these established measurement techniques, we defined the anterior tibial cortex (ATC), tibial proximal anatomical axis (TPAA), and posterior tibial cortex (PTC) as the primary reference axes for our study (Figure 1). In this study we measured angle β (in degrees), defined between the tibial plateau tangent and each respective tibial reference axis. The conventional PTS (α) is defined as the angle between the tibial plateau tangent and a line perpendicular to each tibial reference axis; therefore α (PTS) = 90° − β. All statistical comparisons were performed using β (radiograph-based definition) to maintain consistency across methods. α (=90° − β) is provided for clinical interpretability as the conventional PTS definition. For Bland–Altman plots, differences were computed as radiographic measurement − DVT (in degrees).

### 2.4. Measurement of the Resection Tibial Slope in Intraoperatively Obtained Resectates Measured Using Digital Volume Tomography (DVT)

**Figure 2:**
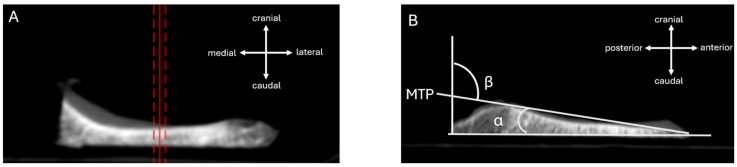
DVT-based measurement of the intraoperatively achieved sagittal tibial slope on the medial tibial resectate. (**A**): Coronal view of the DVT dataset used to standardize specimen orientation and to define the measurement plane (reference line) through the medial resection block, minimizing out-of-plane rotation. The red solid line marks the main reference plane used for the measurement. The red dashed lines are parallel guide lines showing the orientation/centering of that plane. They are not a separate measurement. (**B**): Corresponding sagittal slice in the predefined plane. A tangent is placed along the resection surface (executed tibial cut) and a second tangent along the residual native tibial plateau surface. The tibial slope α is measured as the acute angle between the two tangents; the complement angle is calculated as β = 90° − α.

Following the tibial cut during medial unicompartmental knee arthroplasty, the resected medial tibial bone block preserves the geometry and orientation of the surgeon’s resection plane. Ex vivo DVT of the resectate was therefore used as an executed resection reference for the achieved sagittal alignment, avoiding the projection and overlap limitations of radiographs. First, the DVT volume was reformatted in three orthogonal planes and the specimen was interactively rotated until the resection surface appeared planar and without obliquity in both the coronal and axial views, minimizing out-of-plane rotation. Second, a reproducible sagittal measurement plane was defined through the center of the medial resection block using a reference line on the coronal view (Figure 2A). Third, in the corresponding sagittal slice (Figure 2B), one tangent was placed along the resection surface (executed tibial cut) and a second tangent along the residual native tibial plateau surface. The acute angle between these tangents was defined as the tibial slope (α). For consistency with radiograph-based reporting, the complement angle was calculated as β = 90° − α. In this way, DVT-based resectate analysis provided a direct measure of the executed tibial cut for validating radiograph-derived slope measurements.

All measurements were performed digitally using a DICOM-based image analysis software (MicroDicom^®^,version 2025.1). Each measurement was performed by a single trained observer to ensure consistency.

### 2.5. Statistical Analysis

Qualitative variables were characterized by the frequency of their categories, while quantitative variables were described using mean and standard deviation as well as median with the 1st and 3rd quartiles. Inferential comparisons between the intraoperatively determined tibial slope and those obtained via ATC, TPAA, and PTC were performed using the Wilcoxon signed-rank test, because normality of paired differences could not be assumed (given the sample size and the potential presence of non-Gaussian differences and outliers). No formal multiplicity adjustment was applied, as these hypothesis tests were considered supportive; the primary analysis focused on agreement estimation (Bland–Altman bias and limits of agreement) together with effect sizes and confidence intervals, and *p*-values were interpreted accordingly. To further assess agreement between radiographic measurements (ATC, TPAA, PTC) and the DVT-derived executed resection reference, Bland–Altman analyses were conducted with differences defined as β(method) − β(DVT). Bland–Altman plots were examined for any potential indication of proportional bias (i.e., whether differences might show any systematic trend across the range of tibial slopes). This was considered when interpreting the mean bias as an average measure of agreement across the study sample. In addition to *p*-values, we report standardized effect sizes for paired differences (Cohen’s dz = mean difference/SD of differences) and 95% confidence intervals to facilitate interpretation independent of sample size. All inferential statistical results with *p* < 0.05 were considered statistically significant. All analyses were performed using R (version 4.5.1). Bland–Altman analyses were conducted using the blandr package.

## 3. Results

### 3.1. Demographic Characteristics

A total of 39 patients were included in the study. The mean age was 68.4 ± 8.5 years (range 50–86 years); 23 patients (59.0%) were female and 16 (41.0%) were male.

22 patients (56.4%) underwent navigated UKA and 17 (43.6%) underwent conventional sled-guided UKA. (Table 1).

### 3.2. Comparison of Preoperative Tibial Slope Measurements

The mean intraoperative (DVT) measured angle β was 86.48 ± 1.62° (median 86.4°, range 82.9–89.1°). Using the ATC method, β was 79.69 ± 3.14° (median 78.85°, range 71.64–87.52°). The TPAA method yielded a mean angle of 82.50 ± 2.95° (median 82.24°, range 74.38–88.51°). In contrast, the PTC method produced a mean measured angle of 86.24 ± 2.51° (median 86.79°, range 80.01–89.98°), closely approximating the intraoperatively measured value. The corresponding conventional PTS values were α 3.52° ± 1.62° (DVT), α 10.31° ± 3.14° (ATC), α 7.50° ± 2.95° (TPAA), and α 3.76° ± 2.51° (PTC) (Table 2).

**Figure 3:**
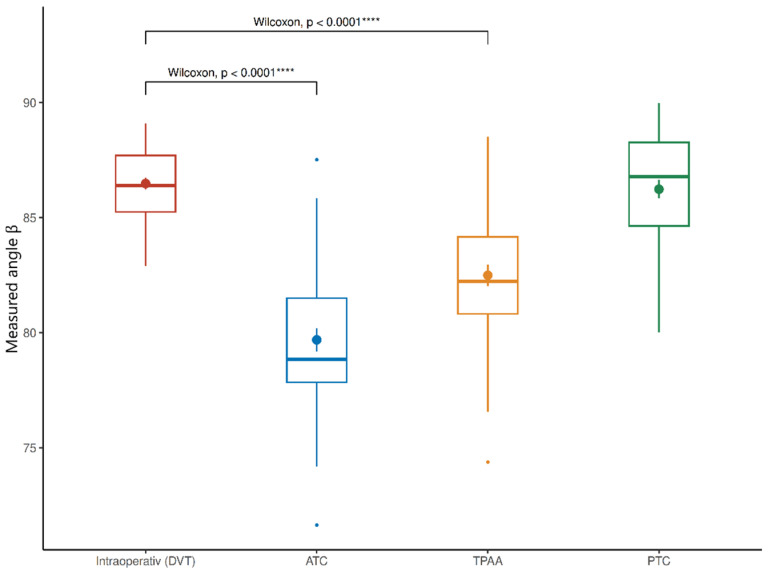
Boxplots comparing intraoperative (DVT-based) measured angle β with preoperative radiographic measurements using ATC, TPAA, and PTC reference axes. **** indicates a statistically significant difference compared with the intraoperative DVT measurement (Wilcoxon signed-rank test, *p* < 0.0001).

Wilcoxon signed-rank testing demonstrated significant differences between the DVT slope and both ATC (*p* < 0.0001) and TPAA (*p* < 0.0001), while no significant difference was observed for PTC (*p* = 0.419) (Figure 3).

To evaluate agreement and systematic bias, Bland–Altman analyses were performed for each reference axis, with differences defined as β(method) − β(DVT).

**Figure 4:**
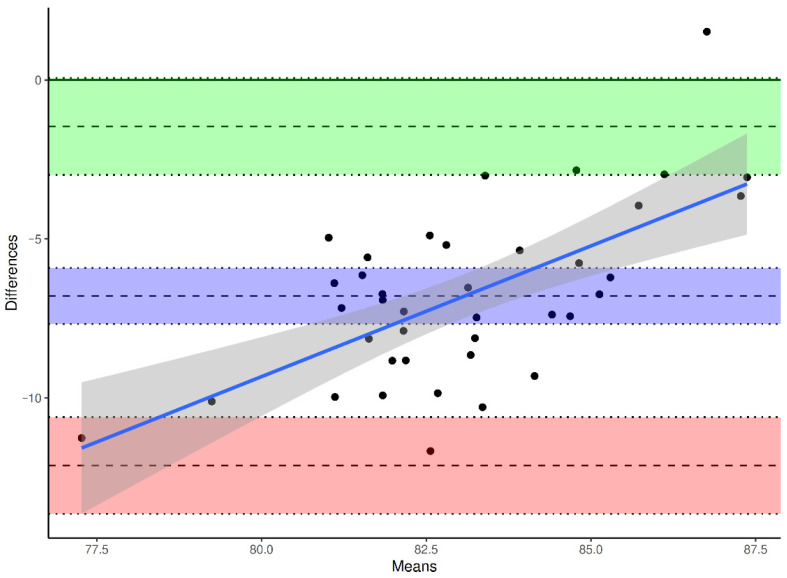
Bland–Altman plot for intraoperative (DVT) vs. ATC-derived preoperative values. Differences are calculated as β(ATC) − β(DVT). Solid line: mean difference (bias). Dashed lines: 95% limits of agreement (mean ± 1.96 SD). Purple indicates the mean bias, green the upper limit of agreement, and red the lower limit of agreement; the shaded bands show the corresponding 95% confidence intervals. The sloping blue line and gray band show the fitted trend and its confidence interval.

**Figure 5:**
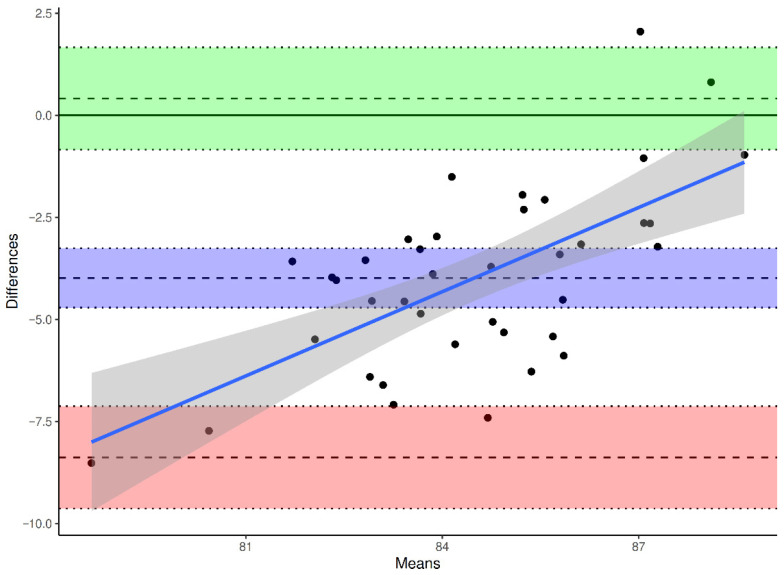
Bland–Altman plot for intraoperative (DVT) vs. TPAA-derived preoperative values. Differences are calculated as β(TPAA) − β(DVT). Solid line: mean difference (bias). Dashed lines: 95% limits of agreement (mean ± 1.96 SD). Purple indicates the mean bias, green the upper limit of agreement, and red the lower limit of agreement; the shaded bands show the corresponding 95% confidence intervals. The sloping blue line and gray band show the fitted trend and its confidence interval.

**Figure 6:**
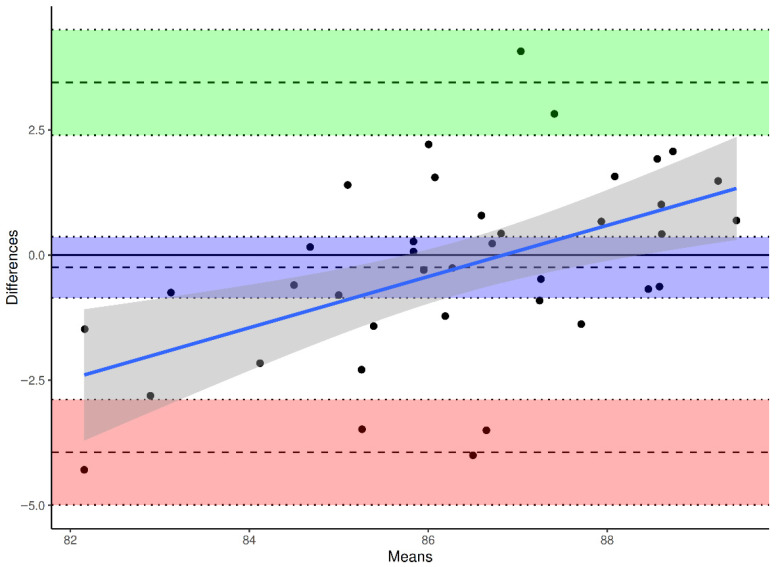
Bland–Altman plot for intraoperative (DVT) vs. PTC-derived preoperative values. Differences are calculated as β(PTC) − β(DVT). Solid line: mean difference (bias). Dashed lines: 95% limits of agreement (mean ± 1.96 SD). Purple indicates the mean bias, green the upper limit of agreement, and red the lower limit of agreement; the shaded bands show the corresponding 95% confidence intervals. The sloping blue line and gray band show the fitted trend and its confidence interval.

The mean bias between ATC and DVT was −6.79° (95% CI −7.67° to −5.91°), the SD of bias was 2.72°, yielding limits of agreement (LoA) between −12.13° and −1.46° (Upper LoA 95% CI −2.99° to 0.06°, Lower LoA 95% CI −13.64° to −10.6°) (Figure 4). The mean bias between TPAA and DVT was –3.99° (95% CI −4.71° to −3.26°), the SD of bias was 2.24°, resulting in LoA from −8.38° to +0.41° (Upper LoA 95% CI −0.84° to 1.66°, Lower LoA 95% CI −9.63° to −7.13°) (Figure 5). The mean bias between PTC and DVT was −0.25° (95% CI −0.86° to +0.36°), the SD of bias was 1.88°, with LoA between −3.94° and +3.45° (Upper LoA 95% CI 2.39° to 4.45°, Lower LoA 95% CI −4.99° to −2.89°) (Figure 6).

Standardized paired effect sizes (Cohen’s dz) based on the Bland–Altman differences were: ATC dz = −2.50, TPAA dz = −1.78, and PTC dz = −0.13. With n = 39, the approximate smallest mean difference detectable with 80% power at α = 0.05 (two-sided) corresponds to ~1.22° (ATC), ~1.00° (TPAA), and ~0.84° (PTC), based on the observed SD of paired differences.

## 4. Discussion

Accurate assessment of the PTS remains central to planning and executing unicompartmental knee arthroplasty (UKA). In this study, the posterior tibial cortex (PTC) method showed the closest agreement with the intraoperatively achieved tibial resection slope measured by DVT of intraoperatively obtained resectates, supporting the view that using an anatomically consistent reference axis reduces variability and improves preoperative precision [30,31,32,33,34].

The anterior tibial cortex (ATC) and tibial proximal anatomical axis (TPAA) methods produced significantly lower values than DVT, corresponding to higher conventional PTS. This pattern aligns with prior work indicating that anterior-based reference definitions are more susceptible to tibial morphology, cortex curvature, and projection-related effects [22,34,35]. Degenerative remodeling and local contour variation may further compromise anterior landmarks, whereas the posterior cortex tends to provide a straighter and more uniform reference, which likely explains the smaller bias observed with PTC.

Inter-individual differences in proximal tibial geometry and compartment-specific slope characteristics can also affect radiographic slope estimation. Advanced imaging studies have reported variability in plateau morphology and medial–lateral slope characteristics, and osteoarthritic changes may alter the apparent slope on conventional imaging [34,35,36].

Because measurement repeatability was not quantified, the Bland–Altman limits of agreement may partly capture reader-dependent measurement error rather than method disagreement, which could widen the limits and affect individual-level conclusions. Single-observer measurements also limit generalizability. Future work should report intra-/interobserver reliability (e.g., ICC, repeatability coefficients).

From a practical standpoint, the closer agreement between PTC and the DVT benchmark suggests that PTC may help reduce systematic under- or over-estimation during preoperative planning. Prior studies have similarly reported smaller observer-related errors and narrower agreement limits for posterior-cortex-derived references compared with alternative approaches [32,33,37]. By improving the fidelity between planned and achieved sagittal alignment, the PTC method may support more consistent intraoperative execution, particularly in settings where the surgeon depends strongly on preoperative imaging to guide slope targets [4,20,21,23].

These measurement differences could have potential clinical relevance because PTS influences UKA biomechanics and has been linked to functional and radiographic outcomes. Excessive posterior slope has been associated with less favorable symptoms and may contribute to instability or component-related problems, whereas slopes within a physiological range have been associated with improved flexion and satisfaction in medial UKA cohorts [14,15,17,36,38]. In this context, a radiographic technique that more closely reflects the executed tibial cut plane could potentially help avoid unintended slope modification and may support more consistent sagittal alignment; however, whether this translates into improved clinical outcomes requires dedicated outcome studies.

Methodologically, DVT assessment of the resected tibial bone block provides a specimen-based reference for the achieved resection plane, minimizing the projection and overlap limitations inherent to radiographs. In our cohort, the PTC-based radiographic approach showed the closest agreement with the executed cut (mean β 86.24° vs. 86.48° for DVT; bias −0.25° with 95% limits of agreement −3.94° to +3.45°), whereas ATC and TPAA showed pronounced systematic underestimation of β (bias −6.79° and −3.99°, respectively), which corresponds to an overestimation of the conventional PTS α by the same magnitude. Similar validation concepts have been applied using CT or EOS-based comparisons [25,34,37]. Although DVT itself is not routinely used for standard clinical planning, its use here strengthens the inference that the observed differences reflect true method-dependent bias rather than measurement noise.

Because the DVT reference is derived from the resected tibial bone block, it represents the executed resection plane and is therefore influenced by intraoperative technique and the alignment philosophy of the instrumentation. In conventional UKA workflows, tibial slope is commonly established using jig design and axis-/landmark-based alignment (e.g., aligning the tibial saw guide with the long axis of the tibia, often with a built-in posterior slope), rather than by posterior cortical alignment. Moreover, there is no consensus on intraoperative references for posterior tibial slope in medial UKA and navigated/robotic workflows may define slope within landmark-based coordinate systems (e.g., transmalleolar axis), which may yield different execution patterns than conventional guides [39,40]. Accordingly, our results should be interpreted as agreement with the performed cut under the present workflow rather than anatomical accuracy of the native posterior tibial slope. If intraoperative technique does not introduce systematic bias and the planned slope target is reproduced accurately, the resected bone block should reflect the intended posterior tibial slope target (i.e., the aimed native slope used for planning), although it does not constitute a direct measure of the preoperative anatomical slope [41].

Although we did not predefine a clinically meaningful threshold for posterior tibial slope differences, interpretation of the absence of a statistically significant difference should rely on the magnitude and precision of the paired difference rather than *p*-values alone. In the present cohort, the mean PTC–DVT difference was small (bias −0.25°), and the 95% confidence interval around this mean difference indicates that systematic discrepancies of approximately 1° or more are unlikely at the group level. However, the Bland–Altman limits of agreement (−3.94° to +3.45°) indicate that individual cases may still show differences in several degrees, and this dispersion should be considered when applying radiographic measurements to single-patient decision-making. Because the DVT reference reflects the executed resection plane and no clinical outcomes were assessed, the clinical relevance of sub-degree differences cannot be inferred from these data.

Finally, the extent to which preoperative slope estimation influences the final result may differ across workflows. Conventional UKA depends more directly on preoperative radiographs and mechanical guides, so systematic preoperative misestimation could translate into unintended changes in gap balancing or joint-line orientation [4,20,21,23]. Navigation and robotic systems may reduce some execution errors, but accurate anatomical referencing remains essential; therefore, more reliable radiographic estimation may still improve planning consistency and facilitate auditing of intended versus achieved alignment [42].

Overall, the closer agreement observed for PTC suggests it may be useful for standardized preoperative planning and postoperative quality-control workflows and may improve reproducibility of planned versus achieved sagittal alignment [15,17,36,38,43].

## 5. Limitations

Several limitations in our study should be acknowledged. The sample size was modest, and the study population was limited to medial UKA cases, which may limit generalizability. Measurements were performed by a single trained observer, and repeat measurements were not performed; therefore, intra- and interobserver reliability could not be quantified. This may influence the precision of agreement estimates (bias and limits of agreement) and limit generalizability to other observers and clinical settings. Radiographs were obtained under controlled conditions in a specialized center; therefore, performance in less standardized environments remains uncertain. The DVT-based resectate approach assumes that specimen handling and fixation do not alter relevant geometry and that the residual native tibial plateau surface can be identified reproducibly on each specimen. Although a standardized reformatting and measurement workflow was applied, subtle deformation and landmark ambiguity may persist and could affect the observed agreement. Furthermore, the study did not evaluate clinical outcomes directly; future research correlating radiographic measurement accuracy with postoperative function, implant longevity, and patient-reported outcomes is warranted.

## 6. Conclusions

Using DVT scans of intraoperatively obtained medial tibial resectates as a specimen-based benchmark for the executed tibial cut plane, this study found that the posterior tibial cortex (PTC) reference axis demonstrated the closest agreement with the intraoperatively achieved tibial resection slope, whereas ATC and TPAA showed systematic deviation. Adoption of the PTC method in preoperative planning could reduce systematic radiographic estimation error in preoperative planning. Whether improved measurement agreement translates into improved intraoperative execution and clinical outcomes should be assessed in prospective studies. Future research should focus on broader clinical validation and integration with automated and AI-supported measurement platforms to standardize PTS assessment across clinical practice.

## Figures and Tables

**Table 1:** Patient demographics and surgical technique (n = 39).

	**Value**
Age (years), mean ± SD (range)	68.4 ± 8.5 (50–86)
Female, n (%)	23 (59.0)
Male, n (%)	16 (41.0)
Navigated UKA, n (%)	22 (56.4)
Conventional sled-guided UKA, n (%)	17 (43.6)

**Table 2:** Radiographic β measurements (ATC, TPAA, PTC) and DVT-derived β from intraoperative tibial resectates. Conventional PTS can be obtained as α = 90° − β. Abbreviations: ATC: anterior tibial cortex; TPAA: tibial proximal anatomical axis; PTC: posterior tibial cortex; DVT: digital volume tomography.

**Reference Method**	**Mean ± SD (°)**	**Median (Q1–Q3)**	**Min–Max (°)**	***p*-Value**
Intraoperative (DVT)	86.48 ± 1.62	86.4 (85.2–87.7)	82.9–89.1	—
ATC	79.69 ± 3.14	78.85 (77.78–81.76)	71.64–87.52	<0.0001
TPAA	82.50 ± 2.95	82.24 (80.65–84.25)	74.38–88.51	<0.0001
PTC	86.24 ± 2.51	86.79 (84.60–88.27)	80.01–89.98	0.419

## Data Availability

The datasets used or analyzed in the current study are available from the corresponding author on reasonable request.
